# 3D-Printed Barrier Membrane Using Mixture of Polycaprolactone and Beta-Tricalcium Phosphate for Regeneration of Rabbit Calvarial Defects

**DOI:** 10.3390/ma14123280

**Published:** 2021-06-14

**Authors:** Jun-Young Lee, Jin-Young Park, In-Pyo Hong, Su-Hee Jeon, Jae-Kook Cha, Jeong-Won Paik, Seong-Ho Choi

**Affiliations:** 1 Department of Periodontology, Research Institute of Periodontal Regeneration, Yonsei University College of Dentistry, Seoul 03722, Korea; juncrazy727@gmail.com (J.-Y.L.); jinyoungpark87@gmail.com (J.-Y.P.); hip0724@gmail.com (I.-P.H.); ssuhee929@yuhs.ac (S.-H.J.); chajaekook@yuhs.ac (J.-K.C.); jpaik@yuhs.ac (J.-W.P.); 2 Innovation Research and Support Center for Dental Science, Yonsei University Dental Hospital, Seoul 03722, Korea

**Keywords:** polycarprolactone, beta tricalcium phosphate, guided bone regeneration, membrane, 3D printing

## Abstract

Background: Polycarprolactone and beta tricalcium phosphate (PCL/β-TCP) are resorbable biomaterials that exhibit ideal mechanical properties as well as high affinity for osteogenic cells. Aim: Objective of this study was to evaluate healing and tissue reaction to the PCL/β-TCP barrier membrane in the rabbit calvaria model for guided bone regeneration. Materials and Methods: The PCL/β-TCP membranes were 3D printed. Three circular defects were created in calvaria of 10 rabbits. The three groups were randomly allocated for each specimen: (i) sham control; (ii) PCL/β-TCP membrane (PCL group); and (iii) PCL/β-TCP membrane with synthetic bone graft (PCL-BG group). The animals were euthanized after two (n = 5) and eight weeks (n = 5) for volumetric and histomorphometric analyses. Results: The greatest augmented volume was achieved by the PCL-BG group at both two and eight weeks (*p* < 0.01). There was a significant increase in new bone after eight weeks in the PCL group (*p* = 0.04). The PCL/β-TCP membrane remained intact after eight weeks with slight degradation, and showed good tissue integration. Conclusions: PCL/β-TCP membrane exhibited good biocompatibility, slow degradation, and ability to maintain space over eight weeks. The 3D-printed PCL/β-TCP membrane is a promising biomaterial that could be utilized for reconstruction of critical sized defects.

## 1. Introduction

Guided bone regeneration (GBR) is a procedure in which a barrier membrane is placed to create a secluded space for the growth of newly mineralized tissue [1]. This procedure is performed in the alveolar crest of patients needing dental implant placement as the supporting bone structures frequently undergo extensive resorption after tooth extraction. The barrier membrane is a critical component of the (GBR) procedure as it separates the defect space from the overlying soft tissues and provides stability for regeneration to occur [1,2]. Until now, numerous non-resorbable and resorbable membranes have been applied with varying degrees of success, and among them, the combination of bone graft particles and collagen membrane is the most commonly used [3]. The collagen membrane is a popular choice as it exhibits excellent biocompatibility and tissue integration. In addition, the collagen is biodegradable; therefore, no additional surgery is needed for membrane removal. However, the collagen membrane lacks some important characteristics such as space maintenance, structural stability, and longevity; which are necessary for the regeneration of severe uncontained defects [4].

For the reconstruction of large defects of the jaw, various options have been considered. Use of numerous fixation pins in combination with collagen membrane and bone graft particles has been suggested previously [5]. Block bone grafts from various sources including autogenous, allogenic, and xenogenic have been utilized [6,7]. Various scaffold materials for bone tissue engineering have been studied as carriers for osteogenic stem cells or growth factors [8,9]. Non-resorbable barrier membranes made of e-PTFE (expanded-polytetrafluoroethylene), d-PTFE (dense-PTFE), and titanium mesh have all been tried due to their superior structural stability and space maintenance [10,11]. However, all of these have drawbacks and complications such as frequent membrane exposure, wound dehiscence, and resulting morbidity of the recipient site [12]. Therefore, there is a tremendous need for a new membrane in this clinical situation that exhibits not only biodegradability, biocompatibility, and tissue integration like the collagen membrane, but also good structural stability and longevity as the non-resorbable membranes.

A synthetic bioresorbable membrane using a combination of polycarprolactone (PCL) and beta tricalcium phosphate (β-TCP) has recently been introduced to overcome the drawbacks of existing membranes [13]. The PCL/β-TCP membrane showed increased mechanical stability and slower degradation compared to conventional collagen membranes with reliable bone regeneration [14]. Simultaneously, the PCL/β-TCP membrane degrades to allow infiltration of cell growth, which induces adequate blending and biocompatible degradation to prevent membrane exposure [15].

Since the PCL/β-TCP membrane is fabricated with 3D printing techniques, the membrane can be designed and printed as an individually tailored membrane, which fits to each bone defect [13]. Acquiring a perfect fit around a bone defect can result in reduced surgery time as well as the prevention of membrane exposure [16]. Thus, due to complementary characteristic of PCL/β-TCP, the 3D printed PCL/β-TCP membrane can simultaneously show similar longevity and mechanical stability of non-resorbable membrane and no necessity of additional surgery [17].

Previous studies have shown the efficacy of PCL/β-TCP membrane in vitro and in vivo [13,14]. However, there was a lack of histologic evaluation in the early healing stage and thorough observation regarding tissue reaction to the PCL/β-TCP membrane per se. Thus, the objective of this study was to evaluate healing and tissue reaction to the PCL/β-TCP membrane in the rabbit calvaria model.

## 2. Materials and Methods

### 2.1. Animals

Ten New Zealand male white rabbits (3~3.5 kg) were prepared for the study. All surgical procedures and animal management were according to the Association for Assessment and Accreditation of Laboratory Animal Care International (AAALAC) guidelines and approved by the Animal Institution Animal Care and Use Committee of Yonsei Medical Center.

### 2.2. Preparation of the PCL and β-TCP Mixture

A PCL/β-TCP membrane (LT6 membrane, Megagen, Daegu, Korea) was used in the study. A detailed manufacturing process has been described in a previous study [18]. In brief, PCL (19561-500G, Polysciences Inc., Warrington, PA, USA) and β-TCP (average diameter: 100 nm, Berkeley Advanced Biomaterials Inc., Berkeley, CA, USA) were blended by a melting process. Inside a glass container, PCL chips were melted to a thermally molten state at 120 °C for 15 min. β-TCP powder was added to the molten PCL and blended together (7:3 ratio of PCL:β-TCP by weight). The molten mixture of PCL and β-TCP were blended for 10 min. 

### 2.3. Fabrication of the PCL/β-TCP Membrane Using a 3D Printer

A micro-extrusion based 3D printer was developed for the fabrication of the PCL/β-TCP membrane, which has been described in detail in a previous study [18]. In brief, the PCL/β-TCP in a molten state (120 °C) was loaded on the multi-head deposition system (MHDS) of the 3D printer. Six-dispensing heads were mounted in the system, which were individually controllable. Four of the heads were connected to a heating system that could melt thermoplastic biomaterials. Melts were dispensed from the nozzle of the 3D printer at 110 °C and 500 kPa. A dispenser that regulates pneumatic pressure was used to extrude the molten biomaterial. The extruded PCL/β-TCP was rapidly cooled and hardened at room temperature. By layer-by-layer, 3D mesh type structure with four layers was printed. Finally, membranes of 30 mm × 20 mm × 0.15 mm size with 250 μm of pore size were produced.

### 2.4. Synthetic Bone Graft Material

Synthetic bone graft material (Bone Matrix I, Megagen, Daegu, Korea) was used in the study. The synthetic bone graft was biphasic calcium phosphate (BCP), which consisted of hydroxyapatite (HA): β-TCP on 60:40 in weight. Pore size of the particle was 100~500 μm for macroporosity and 10~50 μm for microporosity.

### 2.5. Study Design

**Figure 1:**
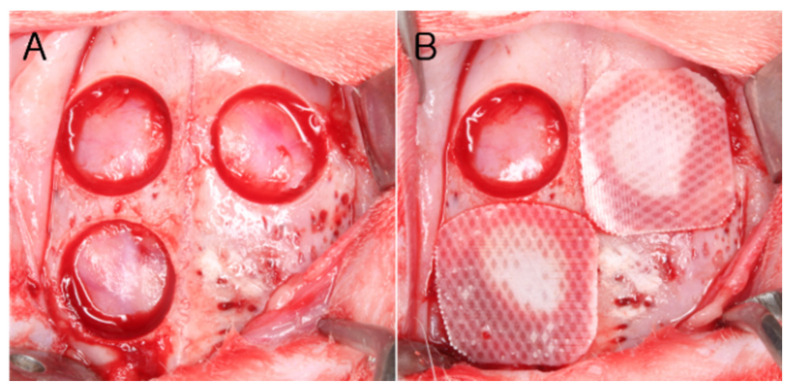
Photograph of the surgical procedure. (**A**) Three round defects with 8-mm diameter were prepared in the calvaria using a trephine bur. (**B**) Each defect was randomly allocated to a study group. Clockwise from bottom left; PCl-BG, control, and PCL.

Ten rabbits were divided into two groups according to healing periods of two and eight weeks, each group containing five rabbits. In the calvarium, three defects having diameter of 8 mm were formed and allocated to the following study groups (Figure 1).

- Sham control group;
- PCL/β-TCP membrane group (PCL group): the defect was covered using the PCL/β-TCP membrane (10 mm × 10 mm) without bone graft; and
- PCL/β-TCP membrane with synthetic bone graft group (PCL-BG group): the defect was filled with synthetic bone graft material and covered using the PCL/β-TCP membrane.

### 2.6. Surgical Procedure

Surgical procedures were described in detail in a previous publication [19]. In brief, surgeries were performed under general anesthesia using inhalation of 2.5% isoflurane and intravenous injection of alfaxan (5 mg/kg). Before incision, surgical sites were disinfected with povidone iodine, and infiltration anesthesia was carried out using 2% lidocaine with 1:80,000 adrenaline. The incision was made along the midline of the calvarium from the frontal bone to the occipital bone, and a full-thickness flap was raised. Three circular defects were made with a trephine bur of 8 mm inner-diameter and each defect was treated as allocated by the study design. Flaps were repositioned and sutured with resorbable suture material (4-0 Vicryl, Ethicon, Somerville, NJ, USA). Sutures were removed one week after surgery. After two or eight weeks of healing, rabbits were sacrificed as allocated and specimens were obtained. 

### 2.7. Data Analysis

#### 2.7.1. Clinical Observations 

The surgical sites were observed for any signs of inflammation and adverse reaction every day until the rabbit was sacrificed. 

#### 2.7.2. Micro-Computed Tomography (Micro-CT) Analysis

The samples were harvested and fixed in 10% formalin over 10 days. They were scanned using a micro-computed tomography (μCT) system (Sky-Scan 1173, SkyScan, Aartselaar, Belgium). The imaging resolution was 13.85 μm (130 kV, 60 μA). The files were processed in DICOM (Digital Imaging and Communications in Medicine) format and reconstructed using On-Demand 3-dimensional (3D) software (Cybermed, Seoul, Korea). For volumetric analysis of the grafted region, the ROImc (region of interest micro-CT) was defined as: the defect margins, laterally; dura mater, inferiorly; and connective tissue border, superiorly. Within the ROImc, the analyzed parameters were:

- Total augmented volume (TAV): the volume of the entire grafted materials and regenerated tissue.
- New bone volume (NBV): the volume of newly formed bone.

For the volumetric analyses of the above parameters, NRecon 1.6.9.8 software was used (Skyscan). Specific 8-bit threshold grayscale values were used for distinguishing the newly formed bone from residual bone substitute. For newly formed bone, the grayscale values were set from 72 to 120 and for bone substitute from 120 to 255.

#### 2.7.3. Histologic Analysis

Following the micro-CT scan, the fixed samples were decalcified in 5% formic acid for 10 days and then embedded in paraffin. The grafted sites were sectioned through the central portion, and 5 μm thick sections were obtained from the middle of the defect. The selected sections were stained with hematoxylin and eosin and Masson trichrome. Observations of the various cells (inflammatory cells, osteoblasts, osteocytes osteoclasts), vasculature, tissues (bone, loose connective tissue), materials (bone substitute, remaining membrane) inside the defect were made using a light microscope (DM LB, Leica Microsystems, Wetzlar, Germany) equipped with a camera (BX50, Olympus, Tokyo, Japan). After the examination, computer-aided organizational measurements were performed for histomorphometric analysis (Photoshop CS6; Adobe Systems, San Jose, CA, USA).

ROIh (region of interest, histologic) was defined by the original defect margin, laterally; dura mater, inferiorly; the periosteum or subcutaneous tissues, superiorly. Within the ROIh, the following parameters were measured:

- Total augmented area (TAA): the area of entire augmented tissues consisting of bone substitute, newly formed bone, loose fibrous connective tissue, and adipose tissue.
- New bone area (NBA): the area of newly formed bone, which can be immature woven bone or mature lamellar bone.

### 2.8. Statistical Analysis

Statistical analysis was performed using a commercial SPSS software program (IBM SPSS Statistics 23; SPSS, Chicago, IL, USA). The Kruskal–Wallis test was used for comparisons between groups at each time point, and the Mann–Whitney U test was used to evaluate the measured parameters between each experimental group. Data are expressed as mean ± standard deviation values with a statistical significance level of 5% (*p* < 0.05). 

## 3. Results

### 3.1. Clinical Observation

No adverse reactions including bleeding and swelling were found after surgery. In addition, the surgical sites healed well without infection or flap exposure. All animals remained healthy throughout the experimental period. 

### 3.2. Micro-CT Analysis

**Figure 2:**
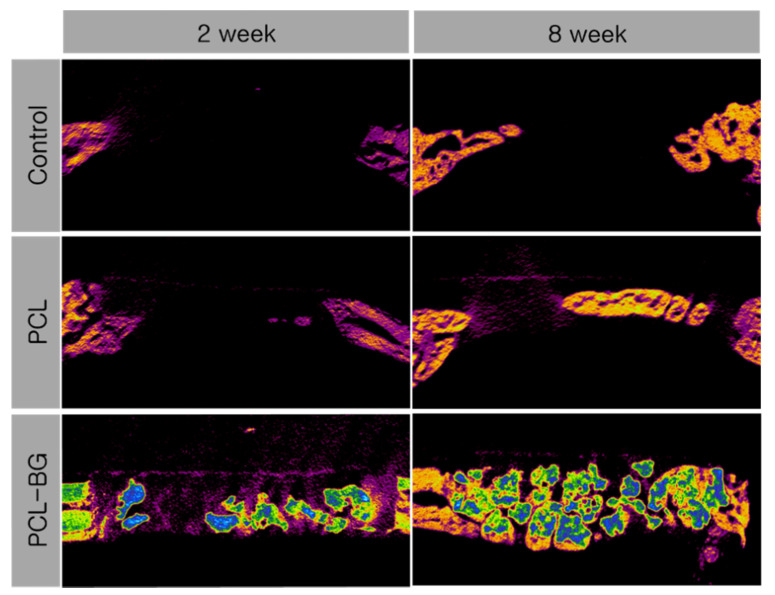
Micro-computed tomographic view of each group at two and eight weeks. Bone is shown in purple, bone substitute particles are shown in yellow-blue. Control group at eight weeks: new bone formation originated from the native marginal bone. PCL group at eight weeks: bone formation was visible in the middle third of the defect as the defect space was maintained by the PCL/β-TCP membrane.

The results of the volumetric analysis are presented in Table 1. Representative view of the micro-CT analysis is shown in Figure 2. At two and eight weeks postoperatively, the PCL-BG group showed significantly greater TBV and NBV compared to the control and PCL groups (*p* = 0.008). Between two and eight weeks, only the PCL group showed a significant increase in NBV (*p* = 0.043).

### 3.3. Observational Histology

**Figure 3:**
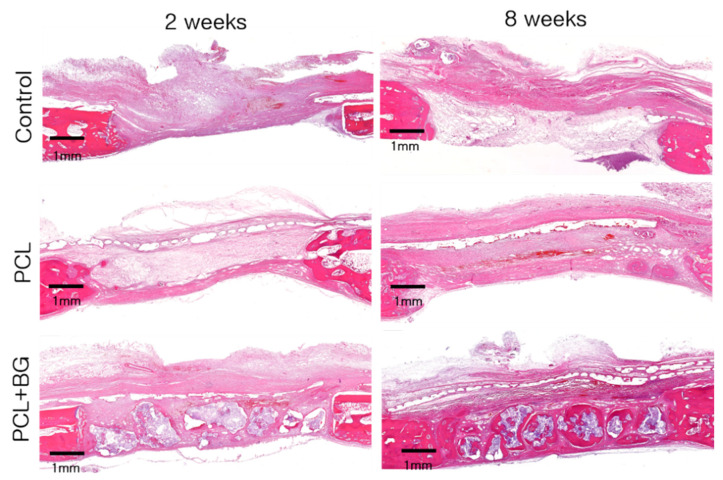
Histological view of each group at two and eight weeks. After eight weeks, the PCL/β-TCP membrane remained intact. Minimal inflammatory reaction was observed. The PCL-BG groups showed the greatest augmented area and new bone formation after eight weeks (H&E staining, scale bar = 1 mm).

Figure 3 shows the histological view of each group at two and eight weeks.

#### 3.3.1. Control Group

In the control group, at two weeks, small areas of new bone formation were found at the margins of the defect. Overlying soft tissues were collapsed into the defect. Inflammatory cells were observed. 

At eight weeks, new bone formation could be observed near the center of the defect. These findings were similar to the sham control group shown in previous studies using the same experimental model [2].

#### 3.3.2. PCL Group 

At two weeks, new bone formation could be observed from the margins of the defect. No inflammatory cells could be observed beneath the membrane. Only a few inflammatory cells were found above the membrane. No collapse of the PCL/β-TCP membrane was seen, and the defect space was well-maintained. 

At eight weeks, the PCL/β-TCP membrane still maintained its shape and thickness. The membrane showed good integration with the surrounding tissues. Some parts of the membrane were resorbed and replaced by connective tissues and new bone. No inflammatory cells were observed. Compared to week 2, more mature lamellar bone could be observed. The structural stability of the PCL/β-TCP membrane was comparable to a previous study in dogs, in which the membrane retained its original shape after eight weeks [17].

#### 3.3.3. PCL-BG Group

At two weeks, new bone formation could be seen originating from the margins of the defect and around the graft particles. The defect space was well maintained by the graft particles and PCL/β-TCP membrane. 

**Figure 4:**
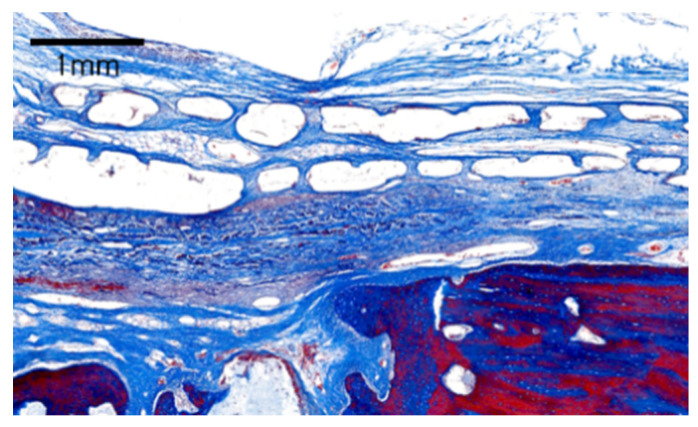
High magnification view showing slight degradation of the membrane after eight weeks. PCL/β-TCP membrane exhibiting a porous appearance with the integration of connective tissue and early new bone (Masson trichrome staining).

At eight weeks, mature new bone formation could be observed across the entire defect, and no inflammatory cells were observed. The PCL/β-TCP membrane remained intact and was integrated with the surrounding tissues (Figure 4). Similar observations were made with regard to the structural integrity and biocompatibility in a previous study in dogs at eight weeks [17].

### 3.4. Histomorphometric Analysis 

The results from the histomorphometric analysis are presented in Table 2. At two weeks, the PCL-BG group showed significantly greater TAA than the control group (*p* = 0.016). At eight weeks, the PCL-BG group exhibited greater TAA than the control and PCL groups (*p* = 0.008). Between two and eight weeks, among the PCL group, NBA was greater at eight weeks compared to two weeks (*p* = 0.043). In addition, among the PCL-BG group, NBA was greater at eight weeks compared to two weeks (*p* = 0.043).

## 4. Discussion

In this study, we evaluated a PCL/β-TCP barrier membrane fabricated using a 3D printer for GBR of critical sized defects in rabbit calvaria [20]. The main findings were: (i) the augmented volume was maximized when the membrane was used with bone graft; (ii) it was possible to gain augmented volume over eight weeks by applying the membrane alone; and (iii) the PCL/β-TCP membrane was highly biocompatible and demonstrated good tissue integration.

Space maintenance is possibly one of the most important requirements of GBR [21]. In dentistry, GBR is performed to increase the volume of alveolar ridge for the placement of dental implants [22]. In order to achieve this, the grafted volume must be maintained over the healing period required for the newly formed bone to fill the space and eventually replace the graft material [23]. In this study, the PCL/β-TCP membrane combined with synthetic bone graft was able to provide the greatest augmented volume, which was maintained over eight weeks. This result was supported by the histological data, in which optimal bone regeneration could be observed inside the defect. The reason for no significant increase in the volume of new bone might be the grafted particles occupying the defect space even after eight weeks. It has been shown that grafted particles take up to six months to become fully replaced by bone [24], and the presence of graft particles may be beneficial as they help to prevent shrinkage of the graft volume [23,25].

As the PCL/β-TCP membrane in this study exhibited slow degradation rate and good structural stability, a faster resorbing bone substitute with greater osteoinductivity could be used together to produce a better quality of new bone. The synthetic bone substitute in this study comprised a combination of HA and β-TCP at the ratio of 60:40 in weight, respectively. HA is known to be osteoconductive while β-TCP is not only osteoconductive but also osteoinductive [26]. Increasing the ratio of the β-TCP component may increase the rate of new bone formation, but results in faster absorption of the material and decreased total augmented volume. However, the current membrane with superior structural stability could accommodate the use of a bone substitute containing higher β-TCP content, thereby obtaining good quality as well as quantity of regenerated bone. 

In this study, it was evident that the PCL/β-TCP membrane can maintain space without the bone graft, as application of the membrane alone resulted in significant increase of new bone over eight weeks. It could be seen in the histological view that the membrane was able to maintain its original structure without collapsing into the defect space. Traditionally, GBR was performed with non-resorbable membranes as they can maintain the barrier function for a controlled period of time, which can be advantageous for the regeneration of uncontained defects and severely atrophic ridges [1,12]. However, there was a need for a second surgery for membrane removal and risk of complication involving frequent membrane exposure [27,28]. The current PCL/β-TCP membrane exhibited slow degradation rate and sufficient structural stability to provide barrier function for eight weeks. PCL has been shown to have a degradation rate that is slower than the rate of bone regeneration [29]. Furthermore, it was shown to have strong mechanical properties that were maintained even after soaking of the membrane, which can be beneficial for space maintenance and manual handling during surgery [14]. One possible drawback of the PCL/β-TCP membrane might be that it has lower wettability compared to the collagen membrane. Therefore, fixation of the membrane may be necessary for stable positioning onto the grafted site. Further study needs to be performed over a longer healing period to assess the degradation characteristics of the current membrane.

Biocompatibility and tissue integration are important properties of a barrier membrane [30]. These are the main advantages of the collagen membrane over non-resorbable membranes that promote good healing and prevent complications such as membrane exposure [31,32]. In this study, no wound dehiscence or membrane exposure was found during the clinical observation period. The histological view showed only a few inflammatory cells at two weeks and good tissue integration of the membrane as shown by connective tissue growth and intramembranous ossification. These appearances were comparable to the tissue integration of the collagen membrane in previous studies in the rabbit calvaria [2]. In vitro experiments in the literature have shown that the PCL/β-TCP membrane exhibits comparable cytocompatibility and osteogenic differentiation as the collagen membrane. Taken together, the current PCL/β-TCP membrane could be used as an alternative to collagen membranes in situations where greater duration of action and structural rigidity is required.

3D printing of the PCL/β-TCP membrane is a rapid and economical solution that allows diverse variations in shape, porosity, and composition. Design and adaptation of the membrane to specific defect morphology would help to reduce surgical time and complication. This technology could be applied to manufacturing a scaffold that may be used as a vessel for growth factors such as rhBMP-2 in tissue engineering [25,33]. 

3D printing can be used to adjust the porosity and pore size of the membrane, which are associated with the degree of new bone formation and angiogenesis. It has been shown that 30% porosity and 130 μm pore size were the most optimal for new bone formation [13]. Although the PCL/β-TCP membrane in the current study performed well with the pore size of 270 μm, further study is needed to assess the effect of varying pore sizes. 

Variation in the composition of the biomaterial results in the alteration of chemical and mechanical properties of the membrane. The addition of β-TCP per se to PCL improved the biocompatibility and bone formation in vivo [17,34]. Increasing the β-TCP concentration in vitro has been shown to increase the rate of absorption and hydrophilicity, which were associated with greater osteogenic cell viability [34,35]. The current study incorporated a 4:1 ratio of PCL:β-TCP. Further research on the degradation characteristics according to varying concentrations would be useful for achieving the optimal membrane for GBR.

## 5. Conclusions

In summary, the PCL/β-TCP membrane exhibited good structural stability, biocompatibility, and slow degradation. In addition, the greatest augmented volume was produced when the membrane was used with a bone substitute. Future studies should test different bone substitutes complementary to this membrane to find the optimal graft material for producing the best quality of new bone. Since the membrane in the current study remained unresorbed over the study period, a further study with longer healing period should be performed to fully understand its degradation characteristics. Overall, the 3D-printed PCL/β-TCP membrane is a promising biomaterial that could be utilized for the reconstruction of critical sized defects.

## Figures and Tables

**Table 1:** Results from the micro-CT analysis.

Healing Period	Study Group	Total Augmented Volume (TAV)	New Bone Volume (NBV)
2 weeks	CONTROL	3.13 ± 2.13	2.94 ± 2.04
	PCL	3.34 ± 1.88	3.22 ± 1.94
	PCL-BG	28.94 ± 8.15 *^,#^	16.14 ± 8.38 *^,#^
8 weeks	CONTROL	10.89 ± 11.77	10.42 ± 11.59
	PCL	20.41 ± 5.75 ^†^	19.45 ± 5.17 ^†^
	PCL-BG	62.20 ± 21.58 *^,#^	33.55 ± 12.08 *^,#^

Values are presented as mean ± standard deviation. * Statistically significant difference compared to the control group. ^#^ Statistically significant difference compared to the PCL group. ^†^ Statistically significant difference between two and eight weeks.

**Table 2:** Results from the histomorphometric analysis.

Healing Period	Study Group	Total Augmented Area (TAA)	New Bone Area (NBA)
2 weeks	CONTROL	4.89 ± 2.51	0.69 ± 0.27
	PCL	8.12 ± 4.26	1.01 ± 0.67
	PCL-BG	13.09 ± 3.89 *	0.90 ± 0.22
8 weeks	CONTROL	5.02 ± 2.69	0.99 ± 0.64
	PCL	8.13 ± 1.15	2.20 ± 1.03 ^†^
	PCL-BG	14.19 ± 3.92 *^,#^	2.80 ± 1.55 ^†^

Values are presented as mean ± standard deviation. * Statistically significant difference compared to the control group. ^#^ Statistically significant difference compared to the PCL group. ^†^ Statistically significant difference between 2 and 8 weeks.

## Data Availability

Data are contained within the article.
